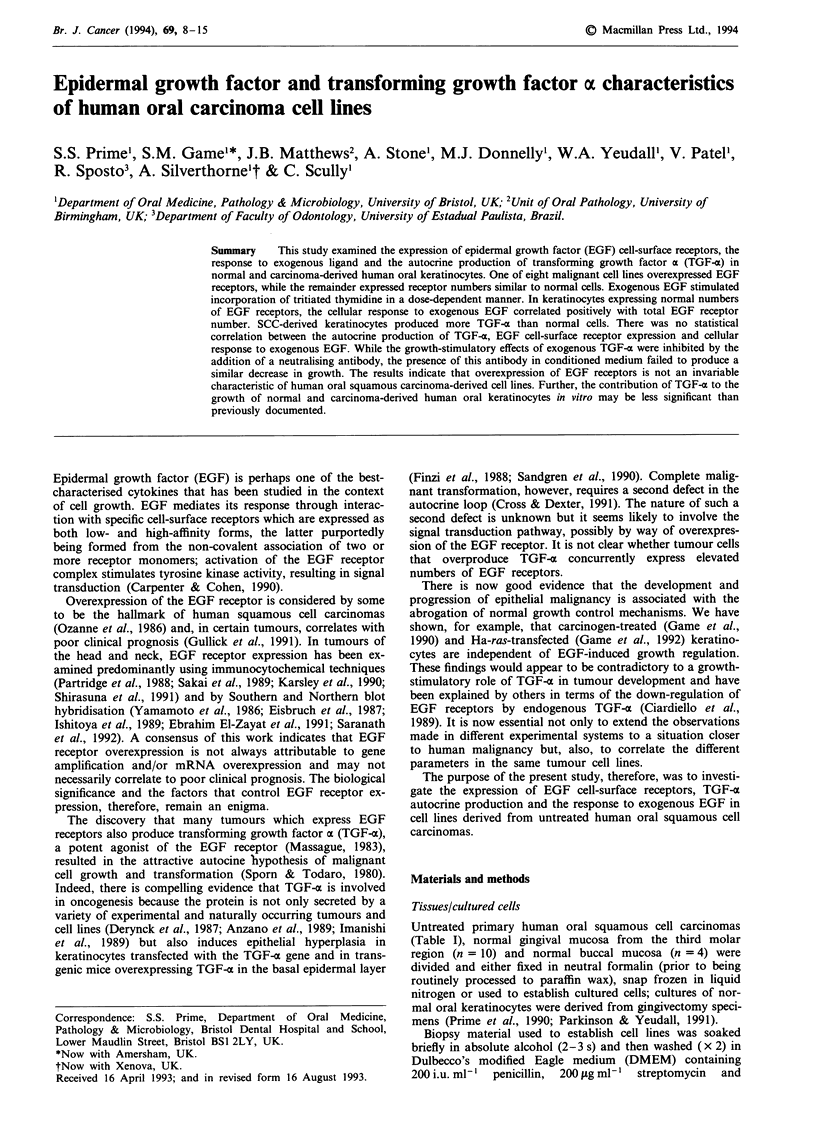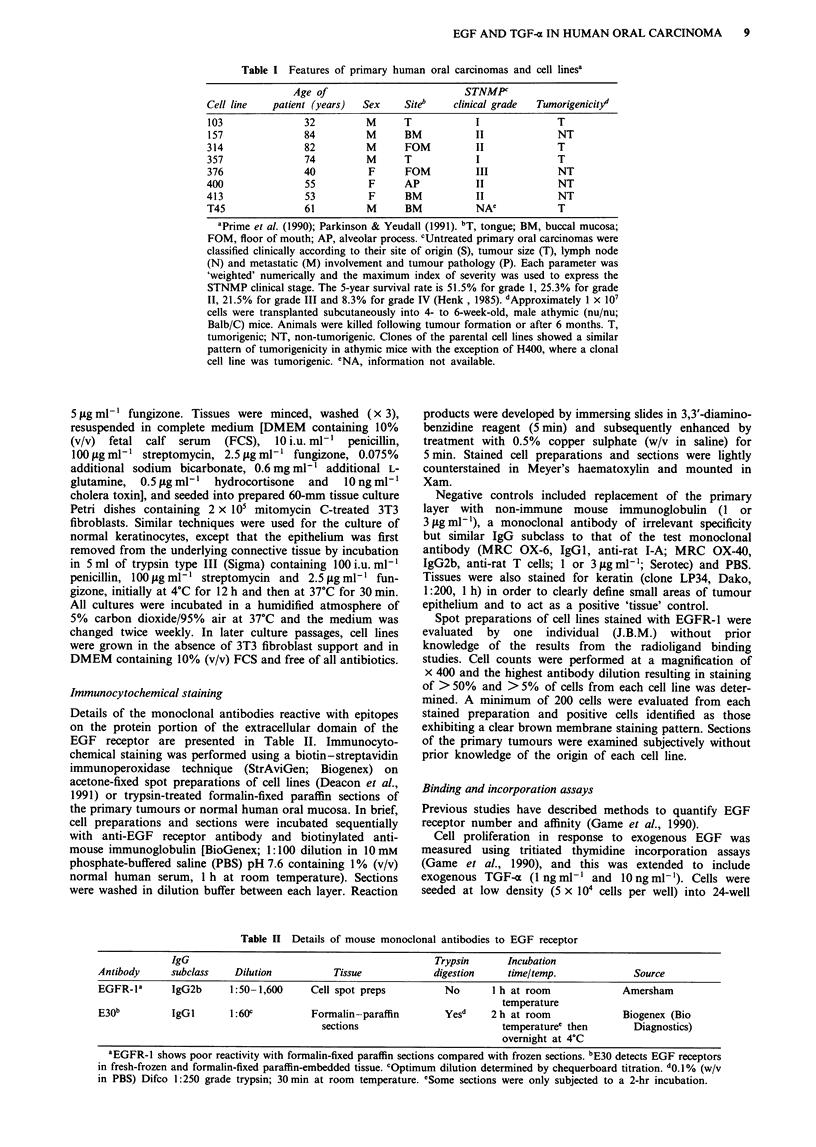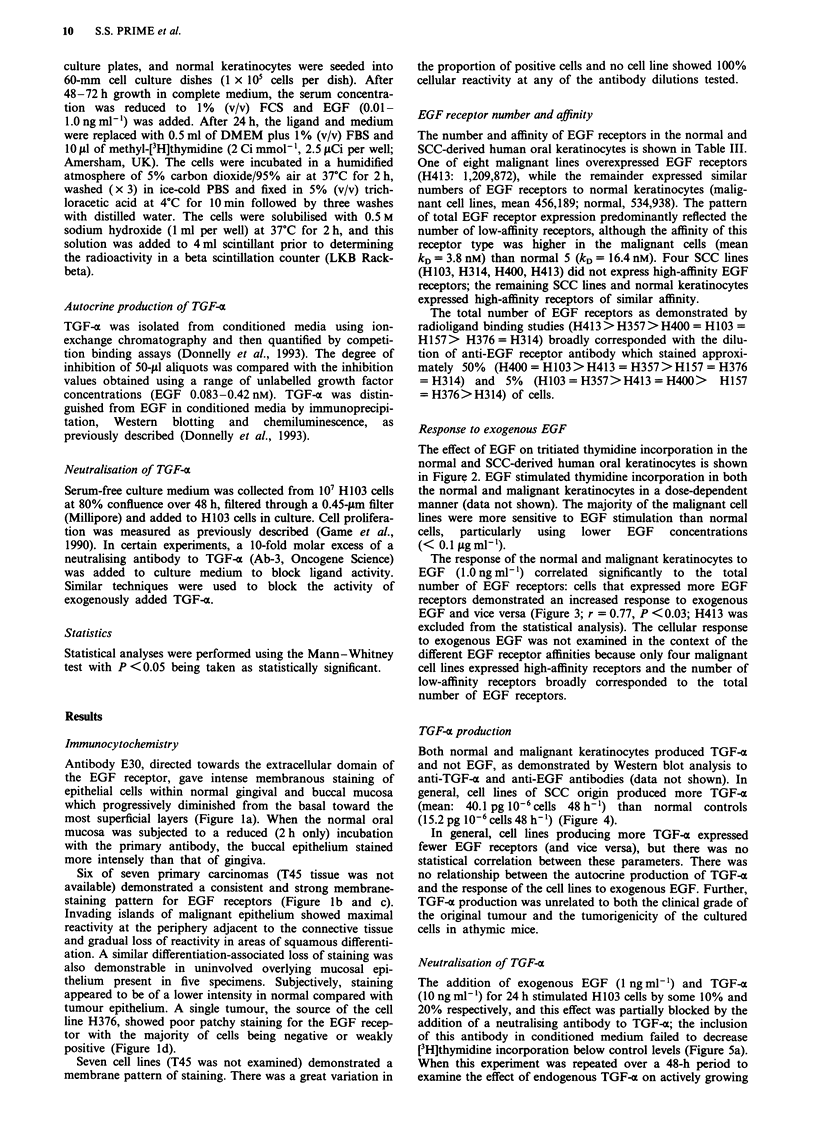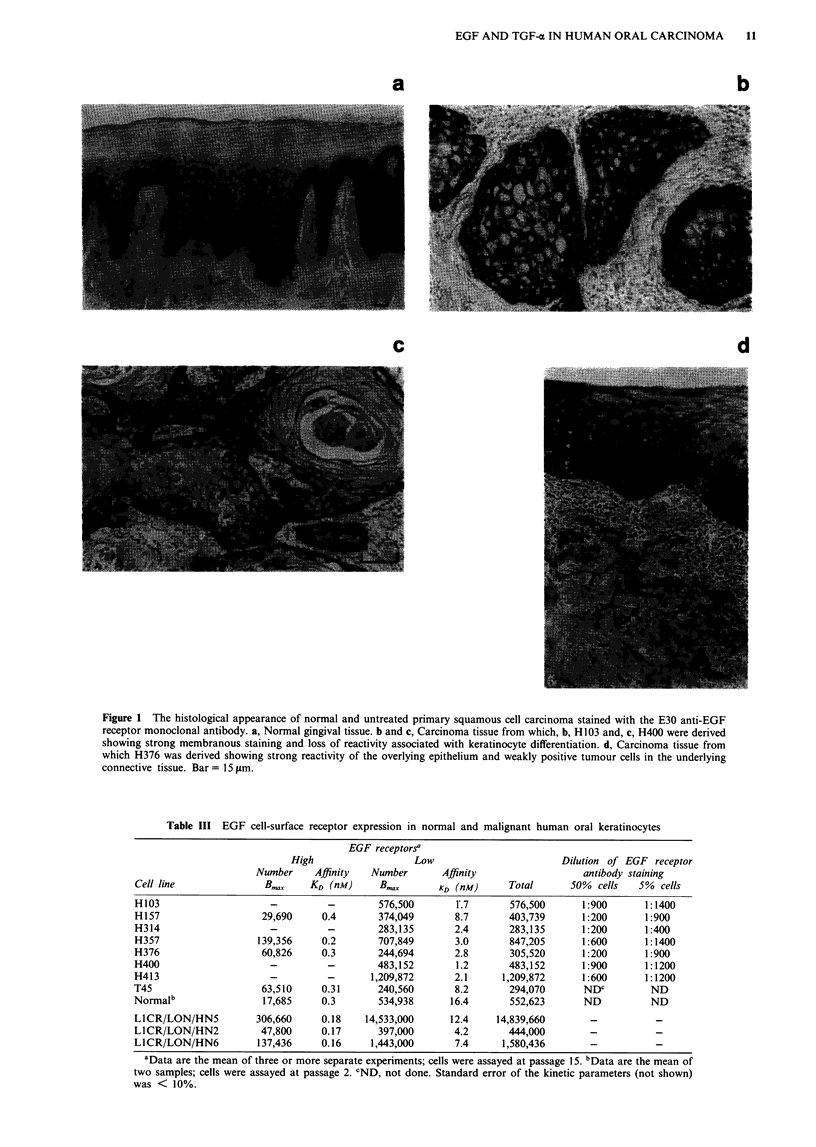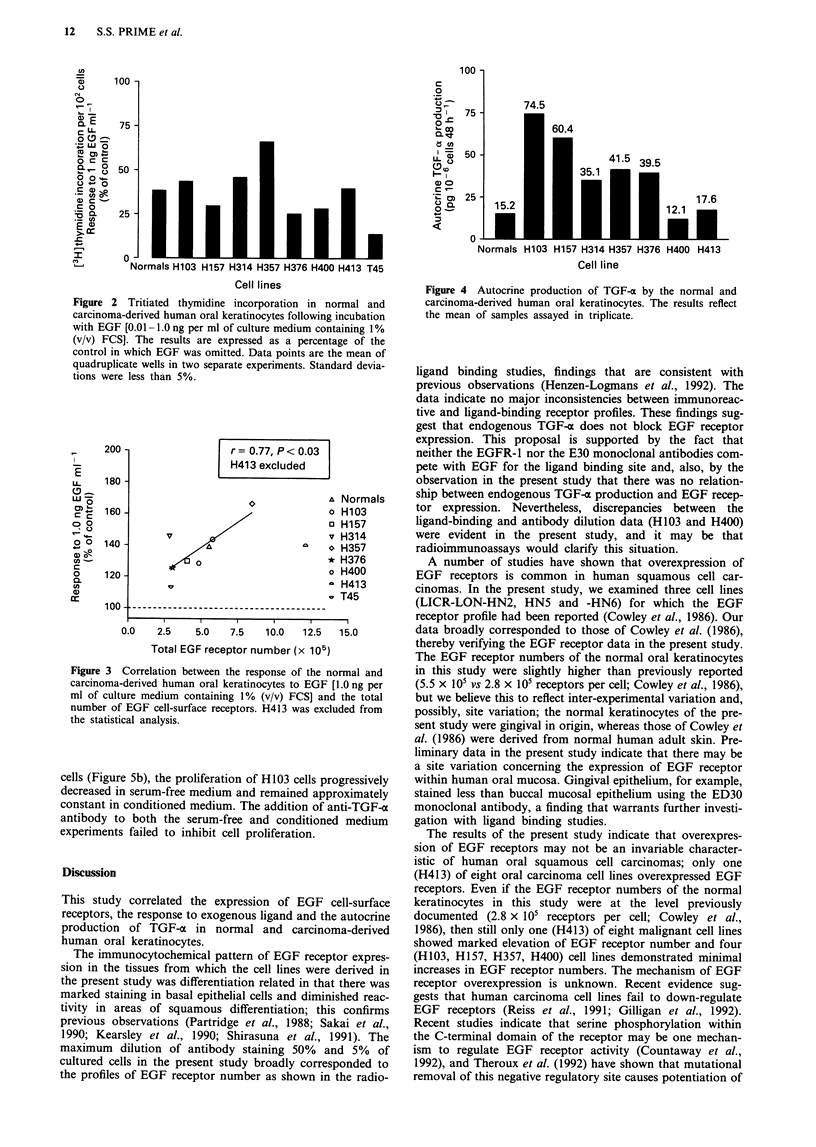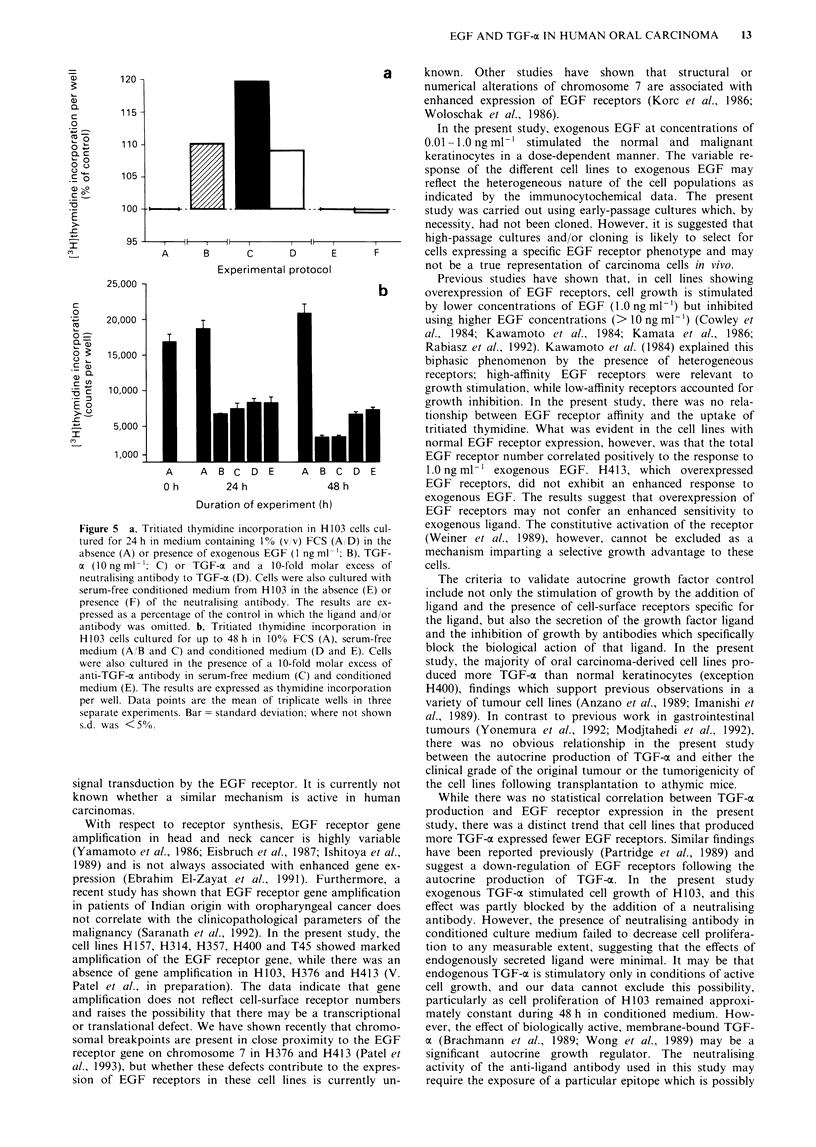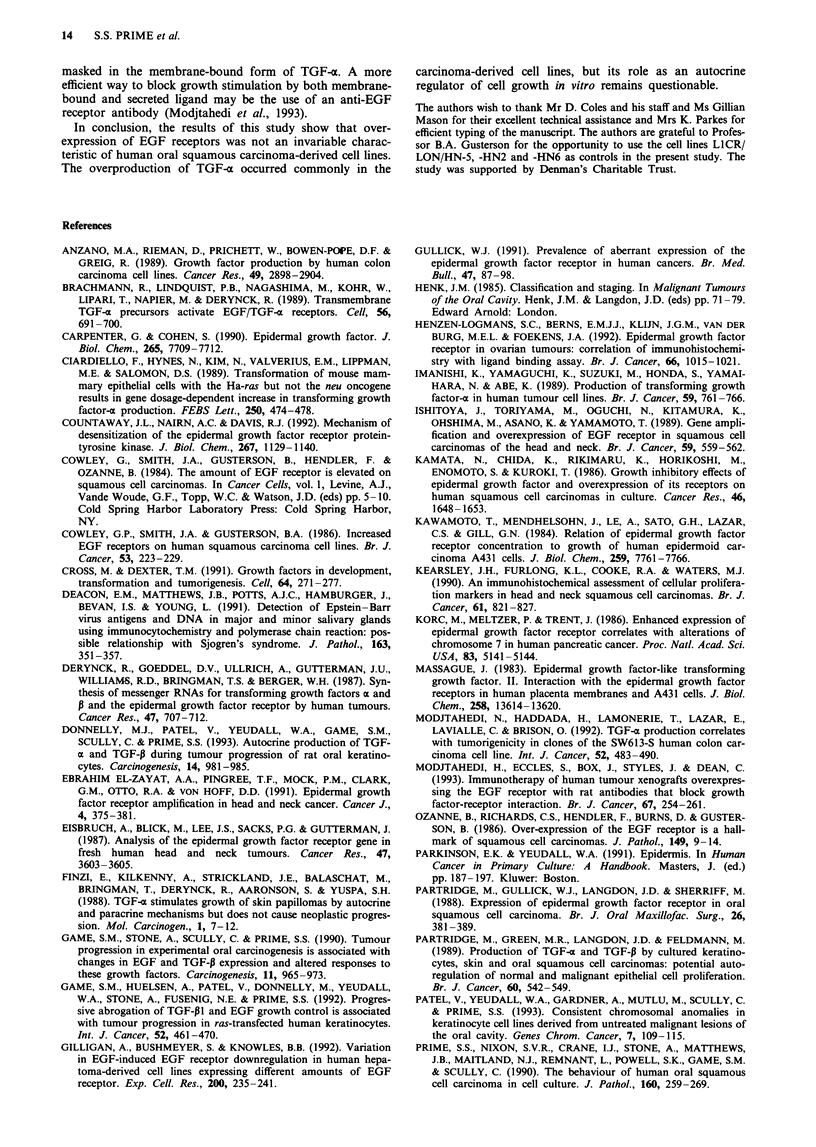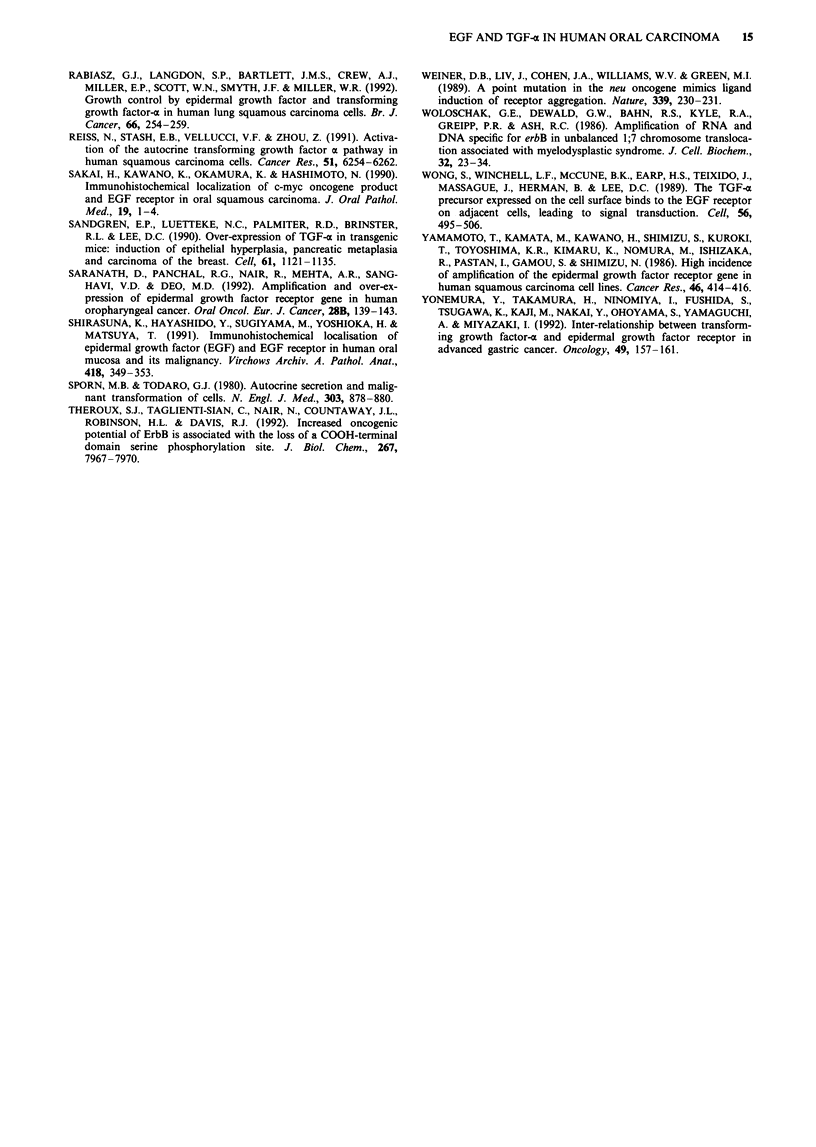# Epidermal growth factor and transforming growth factor alpha characteristics of human oral carcinoma cell lines.

**DOI:** 10.1038/bjc.1994.2

**Published:** 1994-01

**Authors:** S. S. Prime, S. M. Game, J. B. Matthews, A. Stone, M. J. Donnelly, W. A. Yeudall, V. Patel, R. Sposto, A. Silverthorne, C. Scully

**Affiliations:** Department of Oral Medicine, Pathology & Microbiology, University of Bristol, UK.

## Abstract

**Images:**


*Br. J. Cancer* (1994), **69**, 8–15 © Macmillan Press Ltd., 1994

### Epidermal growth factor and transforming growth factor α characteristics of human oral carcinoma cell lines

S.S. Prime[1], S.M. Game[1]*, J.B. Matthews[2], A. Stone[1], M.J. Donnelly[1], W.A. Yeudall[1], V. Patel[1], R. Sposto[3], A. Silverthorne[1]† & C. Scully[1]

[1]*Department of Oral Medicine, Pathology & Microbiology, University of Bristol, UK;* [2]*Unit of Oral Pathology, University of Birmingham, UK;* [3]*Department of Faculty of Odontology, University of Estadual Paulista, Brazil.*

**Summary** This study examined the expression of epidermal growth factor (EGF) cell-surface receptors, the response to exogenous ligand and the autocrine production of transforming growth factor α (TGF-α) in normal and carcinoma-derived human oral keratinocytes. One of eight malignant cell lines overexpressed EGF receptors, while the remainder expressed receptor numbers similar to normal cells. Exogenous EGF stimulated incorporation of tritiated thymidine in a dose-dependent manner. In keratinocytes expressing normal numbers of EGF receptors, the cellular response to exogenous EGF correlated positively with total EGF receptor number. SCC-derived keratinocytes produced more TGF-α than normal cells. There was no statistical correlation between the autocrine production of TGF-α, EGF cell-surface receptor expression and cellular response to exogenous EGF. While the growth-stimulatory effects of exogenous TGF-α were inhibited by the addition of a neutralising antibody, the presence of this antibody in conditioned medium failed to produce a similar decrease in growth. The results indicate that overexpression of EGF receptors is not an invariable characteristic of human oral squamous carcinoma-derived cell lines. Further, the contribution of TGF-α to the growth of normal and carcinoma-derived human oral keratinocytes *in vitro* may be less significant than previously documented.

Epidermal growth factor (EGF) is perhaps one of the best-characterised cytokines that has been studied in the context of cell growth. EGF mediates its response through interaction with specific cell-surface receptors which are expressed as both low- and high-affinity forms, the latter purportedly being formed from the non-covalent association of two or more receptor monomers; activation of the EGF receptor complex stimulates tyrosine kinase activity, resulting in signal transduction (Carpenter & Cohen, 1990).

Overexpression of the EGF receptor is considered by some to be the hallmark of human squamous cell carcinomas (Ozanne *et al.*, 1986) and, in certain tumours, correlates with poor clinical prognosis (Gullick *et al.*, 1991). In tumours of the head and neck, EGF receptor expression has been examined predominantly using immunocytochemical techniques (Partridge *et al.*, 1988; Sakai *et al.*, 1989; Karsley *et al.*, 1990; Shirasuna *et al.*, 1991) and by Southern and Northern blot hybridisation (Yamamoto *et al.*, 1986; Eisbruch *et al.*, 1987; Ishitoya *et al.*, 1989; Ebrahim El-Zayat *et al.*, 1991; Saranath *et al.*, 1992). A consensus of this work indicates that EGF receptor overexpression is not always attributable to gene amplification and/or mRNA overexpression and may not necessarily correlate to poor clinical prognosis. The biological significance and the factors that control EGF receptor expression, therefore, remain an enigma.

The discovery that many tumours which express EGF receptors also produce transforming growth factor α (TGF-α), a potent agonist of the EGF receptor (Massague, 1983), resulted in the attractive autocine hypothesis of malignant cell growth and transformation (Sporn & Todaro, 1980). Indeed, there is compelling evidence that TGF-α is involved in oncogenesis because the protein is not only secreted by a variety of experimental and naturally occurring tumours and cell lines (Derynck *et al.*, 1987; Anzano *et al.*, 1989; Imanishi *et al.*, 1989) but also induces epithelial hyperplasia in keratinocytes transfected with the TGF-α gene and in transgenic mice overexpressing TGF-α in the basal epidermal layer (Finzi *et al.*, 1988; Sandgren *et al.*, 1990). Complete malignant transformation, however, requires a second defect in the autocrine loop (Cross & Dexter, 1991). The nature of such a second defect is unknown but it seems likely to involve the signal transduction pathway, possibly by way of overexpression of the EGF receptor. It is not clear whether tumour cells that overproduce TGF-α concurrently express elevated numbers of EGF receptors.

There is now good evidence that the development and progression of epithelial malignancy is associated with the abrogation of normal growth control mechanisms. We have shown, for example, that carcinogen-treated (Game *et al.*, 1990) and Ha-*ras*-transfected (Game *et al.*, 1992) keratinocytes are independent of EGF-induced growth regulation. These findings would appear to be contradictory to a growth-stimulatory role of TGF-α in tumour development and have been explained by others in terms of the down-regulation of EGF receptors by endogenous TGF-α (Ciardiello *et al.*, 1989). It is now essential not only to extend the observations made in different experimental systems to a situation closer to human malignancy but, also, to correlate the different parameters in the same tumour cell lines.

The purpose of the present study, therefore, was to investigate the expression of EGF cell-surface receptors, TGF-α autocrine production and the response to exogenous EGF in cell lines derived from untreated human oral squamous cell carcinomas.

### Materials and methods

### *Tissues/cultured cells*

Untreated primary human oral squamous cell carcinomas (Table I), normal gingival mucosa from the third molar region ($n = 10$) and normal buccal mucosa ($n = 4$) were divided and either fixed in neutral formalin (prior to being routinely processed to paraffin wax), snap frozen in liquid nitrogen or used to establish cultured cells; cultures of normal oral keratinocytes were derived from gingivectomy specimens (Prime *et al.*, 1990; Parkinson & Yeudall, 1991).

Biopsy material used to establish cell lines was soaked briefly in absolute alcohol (2–3 s) and then washed (× 2) in Dulbecco's modified Eagle medium (DMEM) containing 200 i.u. $ml^{-1}$ penicillin, 200 $\mu g\ ml^{-1}$ streptomycin and

Correspondence: S.S. Prime, Department of Oral Medicine, Pathology & Microbiology, Bristol Dental Hospital and School, Lower Maudlin Street, Bristol BS1 2LY, UK.
*Now with Amersham, UK.
†Now with Xenova, UK.
Received 16 April 1993; and in revised form 16 August 1993.

**Table I** Features of primary human oral carcinomas and cell lines[a]

| Cell line | Age of patient (years) | Sex | Site[b] | STNMP[c] clinical grade | Tumorigenicity[d] |
|---|---|---|---|---|---|
| 103 | 32 | M | T | I | T |
| 157 | 84 | M | BM | II | NT |
| 314 | 82 | M | FOM | II | T |
| 357 | 74 | M | T | I | T |
| 376 | 40 | F | FOM | III | NT |
| 400 | 55 | F | AP | II | NT |
| 413 | 53 | F | BM | II | NT |
| T45 | 61 | M | BM | NA[e] | T |

[a]Prime *et al.* (1990); Parkinson & Yeudall (1991). [b]T, tongue; BM, buccal mucosa; FOM, floor of mouth; AP, alveolar process. [c]Untreated primary oral carcinomas were classified clinically according to their site of origin (S), tumour size (T), lymph node (N) and metastatic (M) involvement and tumour pathology (P). Each parameter was 'weighted' numerically and the maximum index of severity was used to express the STNMP clinical stage. The 5-year survival rate is 51.5% for grade 1, 25.3% for grade II, 21.5% for grade III and 8.3% for grade IV (Henk , 1985). [d]Approximately $1 \times 10^7$ cells were transplanted subcutaneously into 4- to 6-week-old, male athymic (nu/nu; Balb/C) mice. Animals were killed following tumour formation or after 6 months. T, tumorigenic; NT, non-tumorigenic. Clones of the parental cell lines showed a similar pattern of tumorigenicity in athymic mice with the exception of H400, where a clonal cell line was tumorigenic. [e]NA, information not available.

5 μg ml$^{-1}$ fungizone. Tissues were minced, washed (× 3), resuspended in complete medium [DMEM containing 10% (v/v) fetal calf serum (FCS), 10 i.u. ml$^{-1}$ penicillin, 100 μg ml$^{-1}$ streptomycin, 2.5 μg ml$^{-1}$ fungizone, 0.075% additional sodium bicarbonate, 0.6 mg ml$^{-1}$ additional L-glutamine, 0.5 μg ml$^{-1}$ hydrocortisone and 10 ng ml$^{-1}$ cholera toxin], and seeded into prepared 60-mm tissue culture Petri dishes containing $2 \times 10^5$ mitomycin C-treated 3T3 fibroblasts. Similar techniques were used for the culture of normal keratinocytes, except that the epithelium was first removed from the underlying connective tissue by incubation in 5 ml of trypsin type III (Sigma) containing 100 i.u. ml$^{-1}$ penicillin, 100 μg ml$^{-1}$ streptomycin and 2.5 μg ml$^{-1}$ fungizone, initially at 4°C for 12 h and then at 37°C for 30 min. All cultures were incubated in a humidified atmosphere of 5% carbon dioxide/95% air at 37°C and the medium was changed twice weekly. In later culture passages, cell lines were grown in the absence of 3T3 fibroblast support and in DMEM containing 10% (v/v) FCS and free of all antibiotics.

*Immunocytochemical staining*

Details of the monoclonal antibodies reactive with epitopes on the protein portion of the extracellular domain of the EGF receptor are presented in Table II. Immunocytochemical staining was performed using a biotin–streptavidin immunoperoxidase technique (StrAviGen; Biogenex) on acetone-fixed spot preparations of cell lines (Deacon *et al.*, 1991) or trypsin-treated formalin-fixed paraffin sections of the primary tumours or normal human oral mucosa. In brief, cell preparations and sections were incubated sequentially with anti-EGF receptor antibody and biotinylated anti-mouse immunoglobulin [BioGenex; 1:100 dilution in 10 mM phosphate-buffered saline (PBS) pH 7.6 containing 1% (v/v) normal human serum, 1 h at room temperature). Sections were washed in dilution buffer between each layer. Reaction products were developed by immersing slides in 3,3′-diaminobenzidine reagent (5 min) and subsequently enhanced by treatment with 0.5% copper sulphate (w/v in saline) for 5 min. Stained cell preparations and sections were lightly counterstained in Meyer's haematoxylin and mounted in Xam.

Negative controls included replacement of the primary layer with non-immune mouse immunoglobulin (1 or 3 μg ml$^{-1}$), a monoclonal antibody of irrelevant specificity but similar IgG subclass to that of the test monoclonal antibody (MRC OX-6, IgG1, anti-rat I-A; MRC OX-40, IgG2b, anti-rat T cells; 1 or 3 μg ml$^{-1}$; Serotec) and PBS. Tissues were also stained for keratin (clone LP34, Dako, 1:200, 1 h) in order to clearly define small areas of tumour epithelium and to act as a positive 'tissue' control.

Spot preparations of cell lines stained with EGFR-1 were evaluated by one individual (J.B.M.) without prior knowledge of the results from the radioligand binding studies. Cell counts were performed at a magnification of × 400 and the highest antibody dilution resulting in staining of > 50% and > 5% of cells from each cell line was determined. A minimum of 200 cells were evaluated from each stained preparation and positive cells identified as those exhibiting a clear brown membrane staining pattern. Sections of the primary tumours were examined subjectively without prior knowledge of the origin of each cell line.

*Binding and incorporation assays*

Previous studies have described methods to quantify EGF receptor number and affinity (Game *et al.*, 1990).

Cell proliferation in response to exogenous EGF was measured using tritiated thymidine incorporation assays (Game *et al.*, 1990), and this was extended to include exogenous TGF-α (1 ng ml$^{-1}$ and 10 ng ml$^{-1}$). Cells were seeded at low density ($5 \times 10^4$ cells per well) into 24-well

**Table II** Details of mouse monoclonal antibodies to EGF receptor

| Antibody | IgG subclass | Dilution | Tissue | Trypsin digestion | Incubation time/temp. | Source |
|---|---|---|---|---|---|---|
| EGFR-1[a] | IgG2b | 1:50–1,600 | Cell spot preps | No | 1 h at room temperature | Amersham |
| E30[b] | IgG1 | 1:60[c] | Formalin–paraffin sections | Yes[d] | 2 h at room temperature[e] then overnight at 4°C | Biogenex (Bio Diagnostics) |

[a]EGFR-1 shows poor reactivity with formalin-fixed paraffin sections compared with frozen sections. [b]E30 detects EGF receptors in fresh-frozen and formalin-fixed paraffin-embedded tissue. [c]Optimum dilution determined by chequerboard titration. [d]0.1% (w/v in PBS) Difco 1:250 grade trypsin; 30 min at room temperature. [e]Some sections were only subjected to a 2-hr incubation.

culture plates, and normal keratinocytes were seeded into 60-mm cell culture dishes ($1 \times 10^5$ cells per dish). After 48–72 h growth in complete medium, the serum concentration was reduced to 1% (v/v) FCS and EGF (0.01–1.0 ng $ml^{-1}$) was added. After 24 h, the ligand and medium were replaced with 0.5 ml of DMEM plus 1% (v/v) FBS and 10 µl of methyl-[$^3$H]thymidine (2 Ci $mmol^{-1}$, 2.5 µCi per well; Amersham, UK). The cells were incubated in a humidified atmosphere of 5% carbon dioxide/95% air at 37°C for 2 h, washed (× 3) in ice-cold PBS and fixed in 5% (v/v) trichloracetic acid at 4°C for 10 min followed by three washes with distilled water. The cells were solubilised with 0.5 M sodium hydroxide (1 ml per well) at 37°C for 2 h, and this solution was added to 4 ml scintillant prior to determining the radioactivity in a beta scintillation counter (LKB Rackbeta).

*Autocrine production of TGF-α*

TGF-α was isolated from conditioned media using ion-exchange chromatography and then quantified by competition binding assays (Donnelly *et al.*, 1993). The degree of inhibition of 50-µl aliquots was compared with the inhibition values obtained using a range of unlabelled growth factor concentrations (EGF 0.083–0.42 nM). TGF-α was distinguished from EGF in conditioned media by immunoprecipitation, Western blotting and chemiluminescence, as previously described (Donnelly *et al.*, 1993).

*Neutralisation of TGF-α*

Serum-free culture medium was collected from $10^7$ H103 cells at 80% confluence over 48 h, filtered through a 0.45-µm filter (Millipore) and added to H103 cells in culture. Cell proliferation was measured as previously described (Game *et al.*, 1990). In certain experiments, a 10-fold molar excess of a neutralising antibody to TGF-α (Ab-3, Oncogene Science) was added to culture medium to block ligand activity. Similar techniques were used to block the activity of exogenously added TGF-α.

*Statistics*

Statistical analyses were performed using the Mann–Whitney test with $P < 0.05$ being taken as statistically significant.

### Results

*Immunocytochemistry*

Antibody E30, directed towards the extracellular domain of the EGF receptor, gave intense membranous staining of epithelial cells within normal gingival and buccal mucosa which progressively diminished from the basal toward the most superficial layers (Figure 1a). When the normal oral mucosa was subjected to a reduced (2 h only) incubation with the primary antibody, the buccal epithelium stained more intensely than that of gingiva.

Six of seven primary carcinomas (T45 tissue was not available) demonstrated a consistent and strong membrane-staining pattern for EGF receptors (Figure 1b and c). Invading islands of malignant epithelium showed maximal reactivity at the periphery adjacent to the connective tissue and gradual loss of reactivity in areas of squamous differentiation. A similar differentiation-associated loss of staining was also demonstrable in uninvolved overlying mucosal epithelium present in five specimens. Subjectively, staining appeared to be of a lower intensity in normal compared with tumour epithelium. A single tumour, the source of the cell line H376, showed poor patchy staining for the EGF receptor with the majority of cells being negative or weakly positive (Figure 1d).

Seven cell lines (T45 was not examined) demonstrated a membrane pattern of staining. There was a great variation in the proportion of positive cells and no cell line showed 100% cellular reactivity at any of the antibody dilutions tested.

*EGF receptor number and affinity*

The number and affinity of EGF receptors in the normal and SCC-derived human oral keratinocytes is shown in Table III. One of eight malignant lines overexpressed EGF receptors (H413: 1,209,872), while the remainder expressed similar numbers of EGF receptors to normal keratinocytes (malignant cell lines, mean 456,189; normal, 534,938). The pattern of total EGF receptor expression predominantly reflected the number of low-affinity receptors, although the affinity of this receptor type was higher in the malignant cells (mean $k_D = 3.8$ nM) than normal 5 ($k_D = 16.4$ nM). Four SCC lines (H103, H314, H400, H413) did not express high-affinity EGF receptors; the remaining SCC lines and normal keratinocytes expressed high-affinity receptors of similar affinity.

The total number of EGF receptors as demonstrated by radioligand binding studies (H413 > H357 > H400 = H103 = H157 > H376 = H314) broadly corresponded with the dilution of anti-EGF receptor antibody which stained approximately 50% (H400 = H103 > H413 = H357 > H157 = H376 = H314) and 5% (H103 = H357 > H413 = H400 > H157 = H376 > H314) of cells.

*Response to exogenous EGF*

The effect of EGF on tritiated thymidine incorporation in the normal and SCC-derived human oral keratinocytes is shown in Figure 2. EGF stimulated thymidine incorporation in both the normal and malignant keratinocytes in a dose-dependent manner (data not shown). The majority of the malignant cell lines were more sensitive to EGF stimulation than normal cells, particularly using lower EGF concentrations (< 0.1 µg $ml^{-1}$).

The response of the normal and malignant keratinocytes to EGF (1.0 ng $ml^{-1}$) correlated significantly to the total number of EGF receptors: cells that expressed more EGF receptors demonstrated an increased response to exogenous EGF and vice versa (Figure 3; $r = 0.77$, $P < 0.03$; H413 was excluded from the statistical analysis). The cellular response to exogenous EGF was not examined in the context of the different EGF receptor affinities because only four malignant cell lines expressed high-affinity receptors and the number of low-affinity receptors broadly corresponded to the total number of EGF receptors.

*TGF-α production*

Both normal and malignant keratinocytes produced TGF-α and not EGF, as demonstrated by Western blot analysis to anti-TGF-α and anti-EGF antibodies (data not shown). In general, cell lines of SCC origin produced more TGF-α (mean: 40.1 pg $10^{-6}$ cells 48 $h^{-1}$) than normal controls (15.2 pg $10^{-6}$ cells 48 $h^{-1}$) (Figure 4).

In general, cell lines producing more TGF-α expressed fewer EGF receptors (and vice versa), but there was no statistical correlation between these parameters. There was no relationship between the autocrine production of TGF-α and the response of the cell lines to exogenous EGF. Further, TGF-α production was unrelated to both the clinical grade of the original tumour and the tumorigenicity of the cultured cells in athymic mice.

*Neutralisation of TGF-α*

The addition of exogenous EGF (1 ng $ml^{-1}$) and TGF-α (10 ng $ml^{-1}$) for 24 h stimulated H103 cells by some 10% and 20% respectively, and this effect was partially blocked by the addition of a neutralising antibody to TGF-α; the inclusion of this antibody in conditioned medium failed to decrease [$^3$H]thymidine incorporation below control levels (Figure 5a). When this experiment was repeated over a 48-h period to examine the effect of endogenous TGF-α on actively growing

**Figure 1** The histological appearance of normal and untreated primary squamous cell carcinoma stained with the E30 anti-EGF receptor monoclonal antibody. **a**, Normal gingival tissue. **b** and **c**, Carcinoma tissue from which, **b**, H103 and, **c**, H400 were derived showing strong membranous staining and loss of reactivity associated with keratinocyte differentiation. **d**, Carcinoma tissue from which H376 was derived showing strong reactivity of the overlying epithelium and weakly positive tumour cells in the underlying connective tissue. Bar = 15 μm.

**Table III** EGF cell-surface receptor expression in normal and malignant human oral keratinocytes

| | *EGF receptors*[a] | | | | | | |
|---|---|---|---|---|---|---|---|
| | *High* | | *Low* | | | *Dilution of EGF receptor antibody staining* | |
| *Cell line* | *Number* $B_{max}$ | *Affinity* $K_D$ *(nM)* | *Number* $B_{max}$ | *Affinity* $K_D$ *(nM)* | *Total* | *50% cells* | *5% cells* |
| H103 | – | – | 576,500 | 1.7 | 576,500 | 1:900 | 1:1400 |
| H157 | 29,690 | 0.4 | 374,049 | 8.7 | 403,739 | 1:200 | 1:900 |
| H314 | – | – | 283,135 | 2.4 | 283,135 | 1:200 | 1:400 |
| H357 | 139,356 | 0.2 | 707,849 | 3.0 | 847,205 | 1:600 | 1:1400 |
| H376 | 60,826 | 0.3 | 244,694 | 2.8 | 305,520 | 1:200 | 1:900 |
| H400 | – | – | 483,152 | 1.2 | 483,152 | 1:900 | 1:1200 |
| H413 | – | – | 1,209,872 | 2.1 | 1,209,872 | 1:600 | 1:1200 |
| T45 | 63,510 | 0.31 | 240,560 | 8.2 | 294,070 | ND[c] | ND |
| Normal[b] | 17,685 | 0.3 | 534,938 | 16.4 | 552,623 | ND | ND |
| LICR/LON/HN5 | 306,660 | 0.18 | 14,533,000 | 12.4 | 14,839,660 | – | – |
| LICR/LON/HN2 | 47,800 | 0.17 | 397,000 | 4.2 | 444,000 | – | – |
| LICR/LON/HN6 | 137,436 | 0.16 | 1,443,000 | 7.4 | 1,580,436 | – | – |

[a]Data are the mean of three or more separate experiments; cells were assayed at passage 15. [b]Data are the mean of two samples; cells were assayed at passage 2. [c]ND, not done. Standard error of the kinetic parameters (not shown) was $<$ 10%.

**Figure 2** Tritiated thymidine incorporation in normal and carcinoma-derived human oral keratinocytes following incubation with EGF [0.01–1.0 ng per ml of culture medium containing 1% (v/v) FCS]. The results are expressed as a percentage of the control in which EGF was omitted. Data points are the mean of quadruplicate wells in two separate experiments. Standard deviations were less than 5%.

**Figure 3** Correlation between the response of the normal and carcinoma-derived human oral keratinocytes to EGF [1.0 ng per ml of culture medium containing 1% (v/v) FCS] and the total number of EGF cell-surface receptors. H413 was excluded from the statistical analysis.

**Figure 4** Autocrine production of TGF-α by the normal and carcinoma-derived human oral keratinocytes. The results reflect the mean of samples assayed in triplicate.

cells (Figure 5b), the proliferation of H103 cells progressively decreased in serum-free medium and remained approximately constant in conditioned medium. The addition of anti-TGF-α antibody to both the serum-free and conditioned medium experiments failed to inhibit cell proliferation.

### Discussion

This study correlated the expression of EGF cell-surface receptors, the response to exogenous ligand and the autocrine production of TGF-α in normal and carcinoma-derived human oral keratinocytes.

The immunocytochemical pattern of EGF receptor expression in the tissues from which the cell lines were derived in the present study was differentiation related in that there was marked staining in basal epithelial cells and diminished reactivity in areas of squamous differentiation; this confirms previous observations (Partridge *et al.*, 1988; Sakai *et al.*, 1990; Kearsley *et al.*, 1990; Shirasuna *et al.*, 1991). The maximum dilution of antibody staining 50% and 5% of cultured cells in the present study broadly corresponded to the profiles of EGF receptor number as shown in the radioligand binding studies, findings that are consistent with previous observations (Henzen-Logmans *et al.*, 1992). The data indicate no major inconsistencies between immunoreactive and ligand-binding receptor profiles. These findings suggest that endogenous TGF-α does not block EGF receptor expression. This proposal is supported by the fact that neither the EGFR-1 nor the E30 monoclonal antibodies compete with EGF for the ligand binding site and, also, by the observation in the present study that there was no relationship between endogenous TGF-α production and EGF receptor expression. Nevertheless, discrepancies between the ligand-binding and antibody dilution data (H103 and H400) were evident in the present study, and it may be that radioimmunoassays would clarify this situation.

A number of studies have shown that overexpression of EGF receptors is common in human squamous cell carcinomas. In the present study, we examined three cell lines (LICR-LON-HN2, HN5 and -HN6) for which the EGF receptor profile had been reported (Cowley *et al.*, 1986). Our data broadly corresponded to those of Cowley *et al.* (1986), thereby verifying the EGF receptor data in the present study. The EGF receptor numbers of the normal oral keratinocytes in this study were slightly higher than previously reported ($5.5 \times 10^5$ *vs* $2.8 \times 10^5$ receptors per cell; Cowley *et al.*, 1986), but we believe this to reflect inter-experimental variation and, possibly, site variation; the normal keratinocytes of the present study were gingival in origin, whereas those of Cowley *et al.* (1986) were derived from normal human adult skin. Preliminary data in the present study indicate that there may be a site variation concerning the expression of EGF receptor within human oral mucosa. Gingival epithelium, for example, stained less than buccal mucosal epithelium using the ED30 monoclonal antibody, a finding that warrants further investigation with ligand binding studies.

The results of the present study indicate that overexpression of EGF receptors may not be an invariable characteristic of human oral squamous cell carcinomas; only one (H413) of eight oral carcinoma cell lines overexpressed EGF receptors. Even if the EGF receptor numbers of the normal keratinocytes in this study were at the level previously documented ($2.8 \times 10^5$ receptors per cell; Cowley *et al.*, 1986), then still only one (H413) of eight malignant cell lines showed marked elevation of EGF receptor number and four (H103, H157, H357, H400) cell lines demonstrated minimal increases in EGF receptor numbers. The mechanism of EGF receptor overexpression is unknown. Recent evidence suggests that human carcinoma cell lines fail to down-regulate EGF receptors (Reiss *et al.*, 1991; Gilligan *et al.*, 1992). Recent studies indicate that serine phosphorylation within the C-terminal domain of the receptor may be one mechanism to regulate EGF receptor activity (Countaway *et al.*, 1992), and Theroux *et al.* (1992) have shown that mutational removal of this negative regulatory site causes potentiation of

**Figure 5** **a**, Tritiated thymidine incorporation in H103 cells cultured for 24 h in medium containing 1% (v/v) FCS (A/D) in the absence (A) or presence of exogenous EGF (1 ng ml$^{-1}$; B), TGF-α (10 ng ml$^{-1}$; C) or TGF-α and a 10-fold molar excess of neutralising antibody to TGF-α (D). Cells were also cultured with serum-free conditioned medium from H103 in the absence (E) or presence (F) of the neutralising antibody. The results are expressed as a percentage of the control in which the ligand and/or antibody was omitted. **b**, Tritiated thymidine incorporation in H103 cells cultured for up to 48 h in 10% FCS (A), serum-free medium (A/B and C) and conditioned medium (D and E). Cells were also cultured in the presence of a 10-fold molar excess of anti-TGF-α antibody in serum-free medium (C) and conditioned medium (E). The results are expressed as thymidine incorporation per well. Data points are the mean of triplicate wells in three separate experiments. Bar = standard deviation; where not shown s.d. was <5%.

signal transduction by the EGF receptor. It is currently not known whether a similar mechanism is active in human carcinomas.

With respect to receptor synthesis, EGF receptor gene amplification in head and neck cancer is highly variable (Yamamoto *et al.*, 1986; Eisbruch *et al.*, 1987; Ishitoya *et al.*, 1989) and is not always associated with enhanced gene expression (Ebrahim El-Zayat *et al.*, 1991). Furthermore, a recent study has shown that EGF receptor gene amplification in patients of Indian origin with oropharyngeal cancer does not correlate with the clinicopathological parameters of the malignancy (Saranath *et al.*, 1992). In the present study, the cell lines H157, H314, H357, H400 and T45 showed marked amplification of the EGF receptor gene, while there was an absence of gene amplification in H103, H376 and H413 (V. Patel *et al.*, in preparation). The data indicate that gene amplification does not reflect cell-surface receptor numbers and raises the possibility that there may be a transcriptional or translational defect. We have shown recently that chromosomal breakpoints are present in close proximity to the EGF receptor gene on chromosome 7 in H376 and H413 (Patel *et al.*, 1993), but whether these defects contribute to the expression of EGF receptors in these cell lines is currently unknown. Other studies have shown that structural or numerical alterations of chromosome 7 are associated with enhanced expression of EGF receptors (Korc *et al.*, 1986; Woloschak *et al.*, 1986).

In the present study, exogenous EGF at concentrations of 0.01–1.0 ng ml$^{-1}$ stimulated the normal and malignant keratinocytes in a dose-dependent manner. The variable response of the different cell lines to exogenous EGF may reflect the heterogeneous nature of the cell populations as indicated by the immunocytochemical data. The present study was carried out using early-passage cultures which, by necessity, had not been cloned. However, it is suggested that high-passage cultures and/or cloning is likely to select for cells expressing a specific EGF receptor phenotype and may not be a true representation of carcinoma cells *in vivo*.

Previous studies have shown that, in cell lines showing overexpression of EGF receptors, cell growth is stimulated by lower concentrations of EGF (1.0 ng ml$^{-1}$) but inhibited using higher EGF concentrations (> 10 ng ml$^{-1}$) (Cowley *et al.*, 1984; Kawamoto *et al.*, 1984; Kamata *et al.*, 1986; Rabiasz *et al.*, 1992). Kawamoto *et al.* (1984) explained this biphasic phenomenon by the presence of heterogeneous receptors; high-affinity EGF receptors were relevant to growth stimulation, while low-affinity receptors accounted for growth inhibition. In the present study, there was no relationship between EGF receptor affinity and the uptake of tritiated thymidine. What was evident in the cell lines with normal EGF receptor expression, however, was that the total EGF receptor number correlated positively to the response to 1.0 ng ml$^{-1}$ exogenous EGF. H413, which overexpressed EGF receptors, did not exhibit an enhanced response to exogenous EGF. The results suggest that overexpression of EGF receptors may not confer an enhanced sensitivity to exogenous ligand. The constitutive activation of the receptor (Weiner *et al.*, 1989), however, cannot be excluded as a mechanism imparting a selective growth advantage to these cells.

The criteria to validate autocrine growth factor control include not only the stimulation of growth by the addition of ligand and the presence of cell-surface receptors specific for the ligand, but also the secretion of the growth factor ligand and the inhibition of growth by antibodies which specifically block the biological action of that ligand. In the present study, the majority of oral carcinoma-derived cell lines produced more TGF-α than normal keratinocytes (exception H400), findings which support previous observations in a variety of tumour cell lines (Anzano *et al.*, 1989; Imanishi *et al.*, 1989). In contrast to previous work in gastrointestinal tumours (Yonemura *et al.*, 1992; Modjtahedi *et al.*, 1992), there was no obvious relationship in the present study between the autocrine production of TGF-α and either the clinical grade of the original tumour or the tumorigenicity of the cell lines following transplantation to athymic mice.

While there was no statistical correlation between TGF-α production and EGF receptor expression in the present study, there was a distinct trend that cell lines that produced more TGF-α expressed fewer EGF receptors. Similar findings have been reported previously (Partridge *et al.*, 1989) and suggest a down-regulation of EGF receptors following the autocrine production of TGF-α. In the present study exogenous TGF-α stimulated cell growth of H103, and this effect was partly blocked by the addition of a neutralising antibody. However, the presence of neutralising antibody in conditioned culture medium failed to decrease cell proliferation to any measurable extent, suggesting that the effects of endogenously secreted ligand were minimal. It may be that endogenous TGF-α is stimulatory only in conditions of active cell growth, and our data cannot exclude this possibility, particularly as cell proliferation of H103 remained approximately constant during 48 h in conditioned medium. However, the effect of biologically active, membrane-bound TGF-α (Brachmann *et al.*, 1989; Wong *et al.*, 1989) may be a significant autocrine growth regulator. The neutralising activity of the anti-ligand antibody used in this study may require the exposure of a particular epitope which is possibly masked in the membrane-bound form of TGF-α. A more efficient way to block growth stimulation by both membrane-bound and secreted ligand may be the use of an anti-EGF receptor antibody (Modjtahedi *et al.*, 1993).

In conclusion, the results of this study show that over-expression of EGF receptors was not an invariable characteristic of human oral squamous carcinoma-derived cell lines. The overproduction of TGF-α occurred commonly in the carcinoma-derived cell lines, but its role as an autocrine regulator of cell growth *in vitro* remains questionable.

The authors wish to thank Mr D. Coles and his staff and Ms Gillian Mason for their excellent technical assistance and Mrs K. Parkes for efficient typing of the manuscript. The authors are grateful to Professor B.A. Gusterson for the opportunity to use the cell lines L1CR/LON/HN-5, -HN2 and -HN6 as controls in the present study. The study was supported by Denman's Charitable Trust.

### References

ANZANO, M.A., RIEMAN, D., PRICHETT, W., BOWEN-POPE, D.F. & GREIG, R. (1989). Growth factor production by human colon carcinoma cell lines. *Cancer Res.*, **49**, 2898–2904.

BRACHMANN, R., LINDQUIST, P.B., NAGASHIMA, M., KOHR, W., LIPARI, T., NAPIER, M. & DERYNCK, R. (1989). Transmembrane TGF-α precursors activate EGF/TGF-α receptors. *Cell*, **56**, 691–700.

CARPENTER, G. & COHEN, S. (1990). Epidermal growth factor. *J. Biol. Chem.*, **265**, 7709–7712.

CIARDIELLO, F., HYNES, N., KIM, N., VALVERIUS, E.M., LIPPMAN, M.E. & SALOMON, D.S. (1989). Transformation of mouse mammary epithelial cells with the Ha-*ras* but not the *neu* oncogene results in gene dosage-dependent increase in transforming growth factor-α production. *FEBS Lett.*, **250**, 474–478.

COUNTAWAY, J.L., NAIRN, A.C. & DAVIS, R.J. (1992). Mechanism of desensitization of the epidermal growth factor receptor protein-tyrosine kinase. *J. Biol. Chem.*, **267**, 1129–1140.

COWLEY, G., SMITH, J.A., GUSTERSON, B., HENDLER, F. & OZANNE, B. (1984). The amount of EGF receptor is elevated on squamous cell carcinomas. In *Cancer Cells*, vol. 1, Levine, A.J., Vande Woude, G.F., Topp, W.C. & Watson, J.D. (eds) pp. 5–10. Cold Spring Harbor Laboratory Press: Cold Spring Harbor, NY.

COWLEY, G.P., SMITH, J.A. & GUSTERSON, B.A. (1986). Increased EGF receptors on human squamous carcinoma cell lines. *Br. J. Cancer*, **53**, 223–229.

CROSS, M. & DEXTER, T.M. (1991). Growth factors in development, transformation and tumorigenesis. *Cell*, **64**, 271–277.

DEACON, E.M., MATTHEWS, J.B., POTTS, A.J.C., HAMBURGER, J., BEVAN, I.S. & YOUNG, L. (1991). Detection of Epstein–Barr virus antigens and DNA in major and minor salivary glands using immunocytochemistry and polymerase chain reaction: possible relationship with Sjogren's syndrome. *J. Pathol.*, **163**, 351–357.

DERYNCK, R., GOEDDEL, D.V., ULLRICH, A., GUTTERMAN, J.U., WILLIAMS, R.D., BRINGMAN, T.S. & BERGER, W.H. (1987). Synthesis of messenger RNAs for transforming growth factors α and β and the epidermal growth factor receptor by human tumours. *Cancer Res.*, **47**, 707–712.

DONNELLY, M.J., PATEL, V., YEUDALL, W.A., GAME, S.M., SCULLY, C. & PRIME, S.S. (1993). Autocrine production of TGF-α and TGF-β during tumour progression of rat oral keratinocytes. *Carcinogenesis*, **14**, 981–985.

EBRAHIM EL-ZAYAT, A.A., PINGREE, T.F., MOCK, P.M., CLARK, G.M., OTTO, R.A. & VON HOFF, D.D. (1991). Epidermal growth factor receptor amplification in head and neck cancer. *Cancer J.*, **4**, 375–381.

EISBRUCH, A., BLICK, M., LEE, J.S., SACKS, P.G. & GUTTERMAN, J. (1987). Analysis of the epidermal growth factor receptor gene in fresh human head and neck tumours. *Cancer Res.*, **47**, 3603–3605.

FINZI, E., KILKENNY, A., STRICKLAND, J.E., BALASCHAT, M., BRINGMAN, T., DERYNCK, R., AARONSON, S. & YUSPA, S.H. (1988). TGF-α stimulates growth of skin papillomas by autocrine and paracrine mechanisms but does not cause neoplastic progression. *Mol. Carcinogen.*, **1**, 7–12.

GAME, S.M., STONE, A., SCULLY, C. & PRIME, S.S. (1990). Tumour progression in experimental oral carcinogenesis is associated with changes in EGF and TGF-β expression and altered responses to these growth factors. *Carcinogenesis*, **11**, 965–973.

GAME, S.M., HUELSEN, A., PATEL, V., DONNELLY, M., YEUDALL, W.A., STONE, A., FUSENIG, N.E. & PRIME, S.S. (1992). Progressive abrogation of TGF-β1 and EGF growth control is associated with tumour progression in *ras*-transfected human keratinocytes. *Int. J. Cancer*, **52**, 461–470.

GILLIGAN, A., BUSHMEYER, S. & KNOWLES, B.B. (1992). Variation in EGF-induced EGF receptor downregulation in human hepatoma-derived cell lines expressing different amounts of EGF receptor. *Exp. Cell. Res.*, **200**, 235–241.

GULLICK, W.J. (1991). Prevalence of aberrant expression of the epidermal growth factor receptor in human cancers. *Br. Med. Bull.*, **47**, 87–98.

HENK, J.M. (1985). Classification and staging. In *Malignant Tumours of the Oral Cavity*. Henk, J.M. & Langdon, J.D. (eds) pp. 71–79. Edward Arnold: London.

HENZEN-LOGMANS, S.C., BERNS, E.M.J.J., KLIJN, J.G.M., VAN DER BURG, M.E.L. & FOEKENS, J.A. (1992). Epidermal growth factor receptor in ovarian tumours: correlation of immunohistochemistry with ligand binding assay. *Br. J. Cancer*, **66**, 1015–1021.

IMANISHI, K., YAMAGUCHI, K., SUZUKI, M., HONDA, S., YAMAIHARA, N. & ABE, K. (1989). Production of transforming growth factor-α in human tumour cell lines. *Br. J. Cancer*, **59**, 761–766.

ISHITOYA, J., TORIYAMA, M., OGUCHI, N., KITAMURA, K., OHSHIMA, M., ASANO, K. & YAMAMOTO, T. (1989). Gene amplification and overexpression of EGF receptor in squamous cell carcinomas of the head and neck. *Br. J. Cancer*, **59**, 559–562.

KAMATA, N., CHIDA, K., RIKIMARU, K., HORIKOSHI, M., ENOMOTO, S. & KUROKI, T. (1986). Growth inhibitory effects of epidermal growth factor and overexpression of its receptors on human squamous cell carcinomas in culture. *Cancer Res.*, **46**, 1648–1653.

KAWAMOTO, T., MENDHELSOHN, J., LE, A., SATO, G.H., LAZAR, C.S. & GILL, G.N. (1984). Relation of epidermal growth factor receptor concentration to growth of human epidermoid carcinoma A431 cells. *J. Biol. Chem.*, **259**, 7761–7766.

KEARSLEY, J.H., FURLONG, K.L., COOKE, R.A. & WATERS, M.J. (1990). An immunohistochemical assessment of cellular proliferation markers in head and neck squamous cell carcinomas. *Br. J. Cancer*, **61**, 821–827.

KORC, M., MELTZER, P. & TRENT, J. (1986). Enhanced expression of epidermal growth factor receptor correlates with alterations of chromosome 7 in human pancreatic cancer. *Proc. Natl. Acad. Sci. USA*, **83**, 5141–5144.

MASSAGUE, J. (1983). Epidermal growth factor-like transforming growth factor. II. Interaction with the epidermal growth factor receptors in human placenta membranes and A431 cells. *J. Biol. Chem.*, **258**, 13614–13620.

MODJTAHEDI, N., HADDADA, H., LAMONERIE, T., LAZAR, E., LAVIALLE, C. & BRISON, O. (1992). TGF-α production correlates with tumorigenicity in clones of the SW613-S human colon carcinoma cell line. *Int. J. Cancer*, **52**, 483–490.

MODJTAHEDI, H., ECCLES, S., BOX, J., STYLES, J. & DEAN, C. (1993). Immunotherapy of human tumour xenografts overexpressing the EGF receptor with rat antibodies that block growth factor-receptor interaction. *Br. J. Cancer*, **67**, 254–261.

OZANNE, B., RICHARDS, C.S., HENDLER, F., BURNS, D. & GUSTERSON, B. (1986). Over-expression of the EGF receptor is a hallmark of squamous cell carcinomas. *J. Pathol.*, **149**, 9–14.

PARKINSON, E.K. & YEUDALL, W.A. (1991). Epidermis. In *Human Cancer in Primary Culture: A Handbook*. Masters, J. (ed.) pp. 187–197. Kluwer: Boston.

PARTRIDGE, M., GULLICK, W.J., LANGDON, J.D. & SHERRIFF, M. (1988). Expression of epidermal growth factor receptor in oral squamous cell carcinoma. *Br. J. Oral Maxillofac. Surg.*, **26**, 381–389.

PARTRIDGE, M., GREEN, M.R., LANGDON, J.D. & FELDMANN, M. (1989). Production of TGF-α and TGF-β by cultured keratinocytes, skin and oral squamous cell carcinomas: potential autoregulation of normal and malignant epithelial cell proliferation. *Br. J. Cancer*, **60**, 542–549.

PATEL, V., YEUDALL, W.A., GARDNER, A., MUTLU, M., SCULLY, C. & PRIME, S.S. (1993). Consistent chromosomal anomalies in keratinocyte cell lines derived from untreated malignant lesions of the oral cavity. *Genes Chrom. Cancer*, **7**, 109–115.

PRIME, S.S., NIXON, S.V.R., CRANE, I.J., STONE, A., MATTHEWS, J.B., MAITLAND, N.J., REMNANT, L., POWELL, S.K., GAME, S.M. & SCULLY, C. (1990). The behaviour of human oral squamous cell carcinoma in cell culture. *J. Pathol.*, **160**, 259–269.

RABIASZ, G.J., LANGDON, S.P., BARTLETT, J.M.S., CREW, A.J., MILLER, E.P., SCOTT, W.N., SMYTH, J.F. & MILLER, W.R. (1992). Growth control by epidermal growth factor and transforming growth factor-α in human lung squamous carcinoma cells. *Br. J. Cancer*, **66**, 254–259.

REISS, N., STASH, E.B., VELLUCCI, V.F. & ZHOU, Z. (1991). Activation of the autocrine transforming growth factor α pathway in human squamous carcinoma cells. *Cancer Res.*, **51**, 6254–6262.

SAKAI, H., KAWANO, K., OKAMURA, K. & HASHIMOTO, N. (1990). Immunohistochemical localization of c-myc oncogene product and EGF receptor in oral squamous carcinoma. *J. Oral Pathol. Med.*, **19**, 1–4.

SANDGREN, E.P., LUETTEKE, N.C., PALMITER, R.D., BRINSTER, R.L. & LEE, D.C. (1990). Over-expression of TGF-α in transgenic mice: induction of epithelial hyperplasia, pancreatic metaplasia and carcinoma of the breast. *Cell*, **61**, 1121–1135.

SARANATH, D., PANCHAL, R.G., NAIR, R., MEHTA, A.R., SANGHAVI, V.D. & DEO, M.D. (1992). Amplification and over-expression of epidermal growth factor receptor gene in human oropharyngeal cancer. *Oral Oncol. Eur. J. Cancer*, **28B**, 139–143.

SHIRASUNA, K., HAYASHIDO, Y., SUGIYAMA, M., YOSHIOKA, H. & MATSUYA, T. (1991). Immunohistochemical localisation of epidermal growth factor (EGF) and EGF receptor in human oral mucosa and its malignancy. *Virchows Archiv. A. Pathol. Anat.*, **418**, 349–353.

SPORN, M.B. & TODARO, G.J. (1980). Autocrine secretion and malignant transformation of cells. *N. Engl. J. Med.*, **303**, 878–880.

THEROUX, S.J., TAGLIENTI-SIAN, C., NAIR, N., COUNTAWAY, J.L., ROBINSON, H.L. & DAVIS, R.J. (1992). Increased oncogenic potential of ErbB is associated with the loss of a COOH-terminal domain serine phosphorylation site. *J. Biol. Chem.*, **267**, 7967–7970.

WEINER, D.B., LIV, J., COHEN, J.A., WILLIAMS, W.V. & GREEN, M.I. (1989). A point mutation in the *neu* oncogene mimics ligand induction of receptor aggregation. *Nature*, **339**, 230–231.

WOLOSCHAK, G.E., DEWALD, G.W., BAHN, R.S., KYLE, R.A., GREIPP, P.R. & ASH, R.C. (1986). Amplification of RNA and DNA specific for *erb*B in unbalanced 1;7 chromosome translocation associated with myelodysplastic syndrome. *J. Cell. Biochem.*, **32**, 23–34.

WONG, S., WINCHELL, L.F., MCCUNE, B.K., EARP, H.S., TEIXIDO, J., MASSAGUE, J., HERMAN, B. & LEE, D.C. (1989). The TGF-α precursor expressed on the cell surface binds to the EGF receptor on adjacent cells, leading to signal transduction. *Cell*, **56**, 495–506.

YAMAMOTO, T., KAMATA, M., KAWANO, H., SHIMIZU, S., KUROKI, T., TOYOSHIMA, K.R., KIMARU, K., NOMURA, M., ISHIZAKA, R., PASTAN, I., GAMOU, S. & SHIMIZU, N. (1986). High incidence of amplification of the epidermal growth factor receptor gene in human squamous carcinoma cell lines. *Cancer Res.*, **46**, 414–416.

YONEMURA, Y., TAKAMURA, H., NINOMIYA, I., FUSHIDA, S., TSUGAWA, K., KAJI, M., NAKAI, Y., OHOYAMA, S., YAMAGUCHI, A. & MIYAZAKI, I. (1992). Inter-relationship between transforming growth factor-α and epidermal growth factor receptor in advanced gastric cancer. *Oncology*, **49**, 157–161.